# Towards a post-pandemic future for global pathogen genome sequencing

**DOI:** 10.1371/journal.pbio.3002225

**Published:** 2023-08-01

**Authors:** Jason T. Ladner, Jason W. Sahl

**Affiliations:** 1 The Pathogen and Microbiome Institute, Northern Arizona University, Flagstaff, Arizona, United States of America; 2 Department of Biological Sciences, Northern Arizona University, Flagstaff, Arizona, United States of America

## Abstract

Pathogen genome sequencing has become a routine part of our response to active outbreaks of infectious disease and should be an important part of our preparations for future epidemics. In this Essay, we discuss the innovations that have enabled routine pathogen genome sequencing, as well as how genome sequences can be used to understand and control the spread of infectious disease. We also explore the impact of the Severe Acute Respiratory Syndrome Coronavirus 2 (SARS-CoV-2) pandemic on the field of pathogen genomics and outline the challenges we must address to further improve the utility of pathogen genome sequencing in the future.

## Abstract 2

The SARS-CoV-2 pandemic had a major impact on the field of pathogen genomics. This Essay discusses innovations that have enabled routine pathogen genome sequencing, as well as how genome sequences can be used to understand and control the spread of infectious disease and discusses outstanding challenges to improve pathogen genome sequencing in the future.

## Introduction

Less than a century ago, the public health impact of infectious disease was thought to have largely been resolved. By the 1960s, we had a detailed understanding of the various microbes that cause infectious disease: viruses, bacteria, and fungi. We also knew how these pathogens spread and had made extraordinary progress towards the prevention and treatment of infectious disease through the development and use of antibiotics and vaccines, as well as societal changes related to personal hygiene and sanitation [1]. What we did not fully appreciate at the time, however, was the incredible diversity of human pathogens, their capacity for rapid evolution, and the dynamic nature of interactions between pathogens and their hosts. Combined, these factors have substantially complicated our attempts to mitigate the impacts of infectious disease.

One of the major reasons for this is the continued emergence of new pathogens, as well as the reemergence of known pathogens in different forms and/or places. H1N1 influenza virus, human immunodeficiency virus (HIV), and Severe Acute Respiratory Syndrome Coronavirus 2 (SARS-CoV-2) have all emerged relatively recently through zoonotic transmission from animals to humans. We have also repeatedly seen known pathogens reemerge in forms that are difficult or impossible to treat with available drugs. For example, our widespread use of antibiotics has selected for new, multidrug-resistant strains of many bacteria, including *Staphylococcus aureus*, *Escherichia coli*, and *Pseudomonas aeruginosa*. Another reason it has been challenging to mitigate the public health impact of infectious disease is that not all pathogens are easily controlled with existing approaches. Despite our early successes using vaccines to stop the spread of viruses like variola virus and poliovirus, and bacteria like *Bordetella pertussis* and *Clostridium tetani*, other pathogens have been much more difficult to control using vaccines; for example, due to the co-circulation of multiple serotypes and the existence of nonhuman or environmental reservoirs. We have also made great progress in the development of antiviral therapeutics, but in many cases their effectiveness depends on rapid and specific diagnosis, which remains a challenge. Societal changes like increases in population size and density, environmental degradation, and increases in the frequency of long-distance travel, have raised the likelihood of zoonotic transmission and made it easier for pathogens to spread within populations and around the world. In addition, we continue to struggle with public acceptance of existing interventions, which can severely limit their utility.

Fortunately, we have also continued to develop new tools that are allowing us to prepare for and respond to infectious disease outbreaks in more targeted ways, one of them being pathogen genome sequencing [2]. A pathogen’s nucleic acid genome (DNA or RNA) contains all of the information needed for its proper development and function. Therefore, genome sequences can teach us about the biology of pathogens, and they also serve as unique barcodes for pathogen identification and tracking. We can now routinely and cost-effectively generate full-length genome sequences in near real time, even for pathogens with larger genome sizes, like bacteria and fungi. Using these sequences, we can diagnose infectious diseases, learn about the dynamics of pathogen spread, and make informed, patient-level treatment decisions.

In this Essay, we discuss the rapid rise of pathogen genome sequencing, beginning in the 2000s and then accelerating with the emergence and global spread of SARS-CoV-2 in 2019. We start with a discussion of the technological advances that enabled routine pathogen genome sequencing, then describe the various uses of pathogen genomic information for understanding and fighting infectious disease, as well as several of the important advances in this field that were driven by the SARS-CoV-2 pandemic and end with a discussion of the needs and future challenges for pathogen sequencing.

### Enabling routine pathogen sequencing

**Fig 1:**
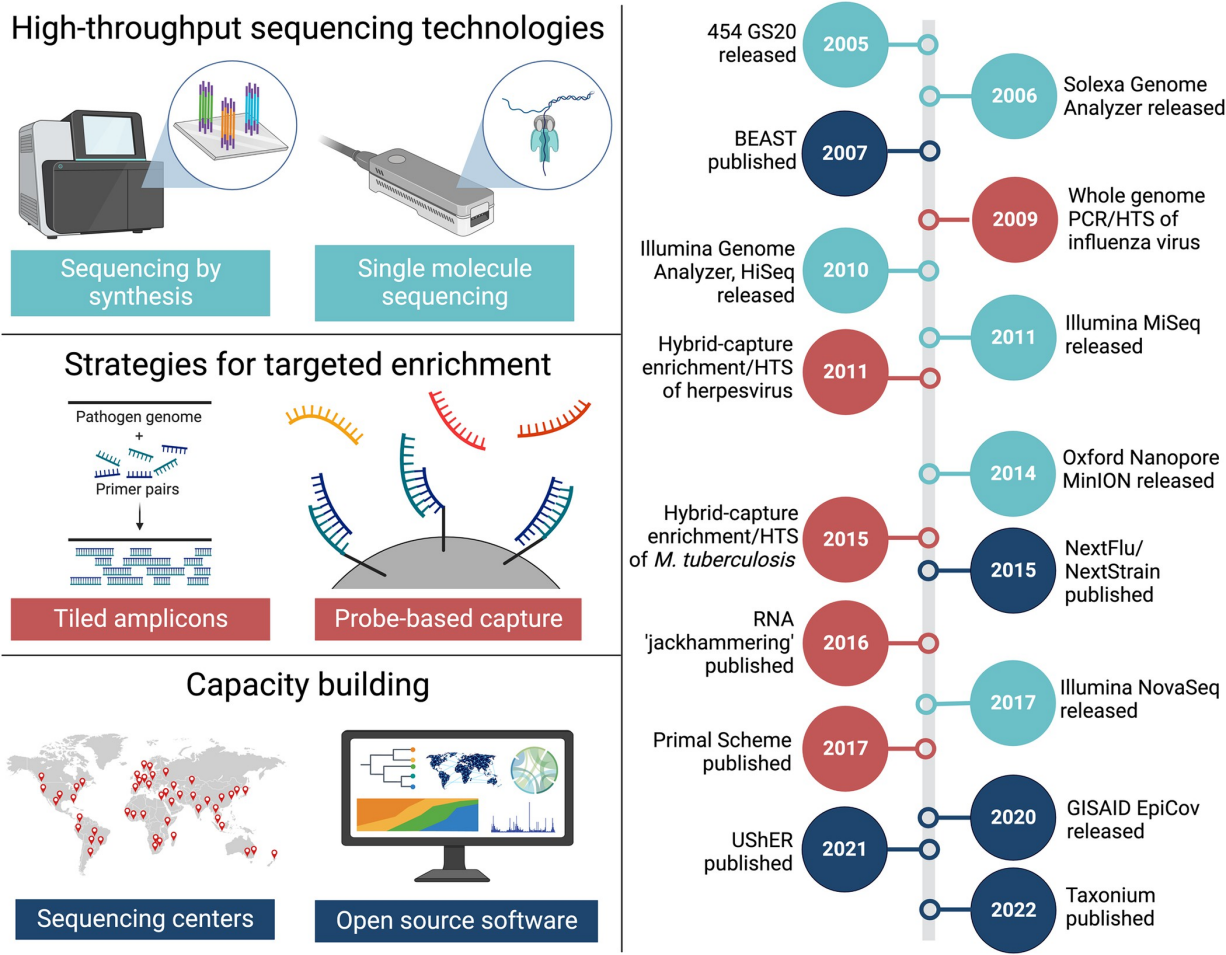
Advances that have enabled routine sequencing of pathogen genomes. Timeline (right) includes a select number of related technology release/publication dates, with colors linking each event to one of 3 general categories of advancement (left). HTS, high-throughput sequencing. Created with BioRender.com.

The utility of genetic data for tracking and understanding pathogens has been recognized for several decades, but routine, full-length genome sequencing has only become possible within the last approximately 10 years thanks to several important technological advances (Fig 1). Without question, the most important of these advances was the development of high-throughput (aka “next-generation”) DNA sequencing. Several approaches for high-throughput sequencing came to market around the same time (2005 to 2007; e.g., 454 [3], Solexa [4], Illumina [5]) and they all enabled, for the first time, massively parallel sequencing of diverse pools of nucleic acids. These technologies enabled genome sequencing by significantly reducing the per base cost of DNA sequencing and providing an efficient approach for sequencing DNA in a nonspecific manner (i.e., not utilizing predefined priming sites). Over the years, incremental improvements in some of these initial technologies (e.g., Illumina’s sequencing by synthesis [6]) have resulted in progressively longer reads, higher throughput, and lower cost. Meanwhile, several new, single molecule sequencing approaches have also been introduced (e.g., Oxford Nanopore Technologies [7]) and these have significantly increased read length (1,000s versus 100s of bases per read), thus facilitating the assembly of larger genomes, while also decreasing the cost and size of the sequencing instruments, thus increasing the accessibility and portability of high-throughput sequencing.

A second, related advance has involved capacity building for the use of high-throughput technologies. Although these technologies initially debuted nearly 2 decades ago, the sequencing hardware and the expertise for running the instruments and interpreting the results were initially concentrated within a small number of labs and almost exclusively within a handful of high-income countries. In contrast, infectious disease outbreaks are a global concern, and many of the recognized hot spots for emerging infectious diseases are within the Global South. This initial discordance between the availability and need for high-throughput technologies complicated timely genomic responses to infectious disease outbreaks, such as the Ebola virus epidemic in West Africa in 2013 to 2016 [8]. Over the intervening years, however, global access to high-throughput sequencing for outbreak responses has grown immensely (though not equally) due to a combination of decreasing costs for sequencing hardware and reagents, dedicated efforts from international agencies and local governments to build sequencing capacity in low-and-middle-income countries (LMICs), the release of open source, freely available software packages and web resources for the analysis and interpretation of pathogen genomes (e.g., BEAST [9], Galaxy [10], NextStrain [11], CZ ID [12]) and the occurrence of multiple outbreaks of international concern, including the Coronavirus Disease 2019 (COVID-19) pandemic. Importantly, there has also been a steady migration of sequencing expertise from industry and academia into the public health laboratories that serve as the first line response to outbreaks of infectious disease.

Another crucial set of advances enabled the enrichment of pathogen-derived nucleic acids from complex samples. Although high-throughput sequencing can deliver full pathogen genomes without targeted enrichment, this is generally not cost effective because pathogen-derived nucleic acids are often present at very low abundance within relevant samples (e.g., blood, feces, saliva, soil, and air filters), and traditional enrichment approaches involving laboratory culturing are time consuming and dependent on the presence of a sufficient number of infectious particles. Therefore, novel approaches for enrichment were needed to enable routine pathogen genome sequencing within clinically relevant time frames. The most successful approaches fall into 2 categories: depletion of nontarget nucleic acids, like host ribosomal RNA, which are often the most abundant RNAs within clinical samples [13], or specific enrichment of pathogen-derived nucleic acids. Two primary methods have been successful for pathogen genome enrichment: selective amplification through PCR and probe-based hybrid-capture (Fig 1). Whole-genome amplification has been used for decades to study RNA viruses like influenza A [14] and HIV [15], and the potential for combining whole-genome amplification with high-throughput sequencing was initially demonstrated with these same viruses [16,17]. In subsequent years, tiled amplicon sequencing has been applied to a wide variety of pathogens, and while most of the initial methods focused on a small number of large amplicons (approximately 1,000 to 3,000 nt), many of the newer methods use highly multiplexed pools of primers that generate short amplicons (approximately 400 nt) and therefore can amplify pathogen genomes even within degraded samples with low titers (e.g., RNA “jackhammering” [18], Primal Scheme [19]). This type of enrichment is relatively cheap and simple to set up, but the primer panels are also highly specific for a particular pathogen and the approach is not easily scalable to larger genomes, such as dsDNA viruses, bacteria, and fungi. In contrast, probe-based, hybrid-capture methods can simultaneously enrich nucleic acids from multiple distinct pathogens and across complete genomes of even the largest infectious agents [20,21]. However, this method is more expensive due largely to the cost of synthesizing the oligonucleotides (i.e., probes) used for selective capture.

## How pathogen genomes are used

**Fig 2:**
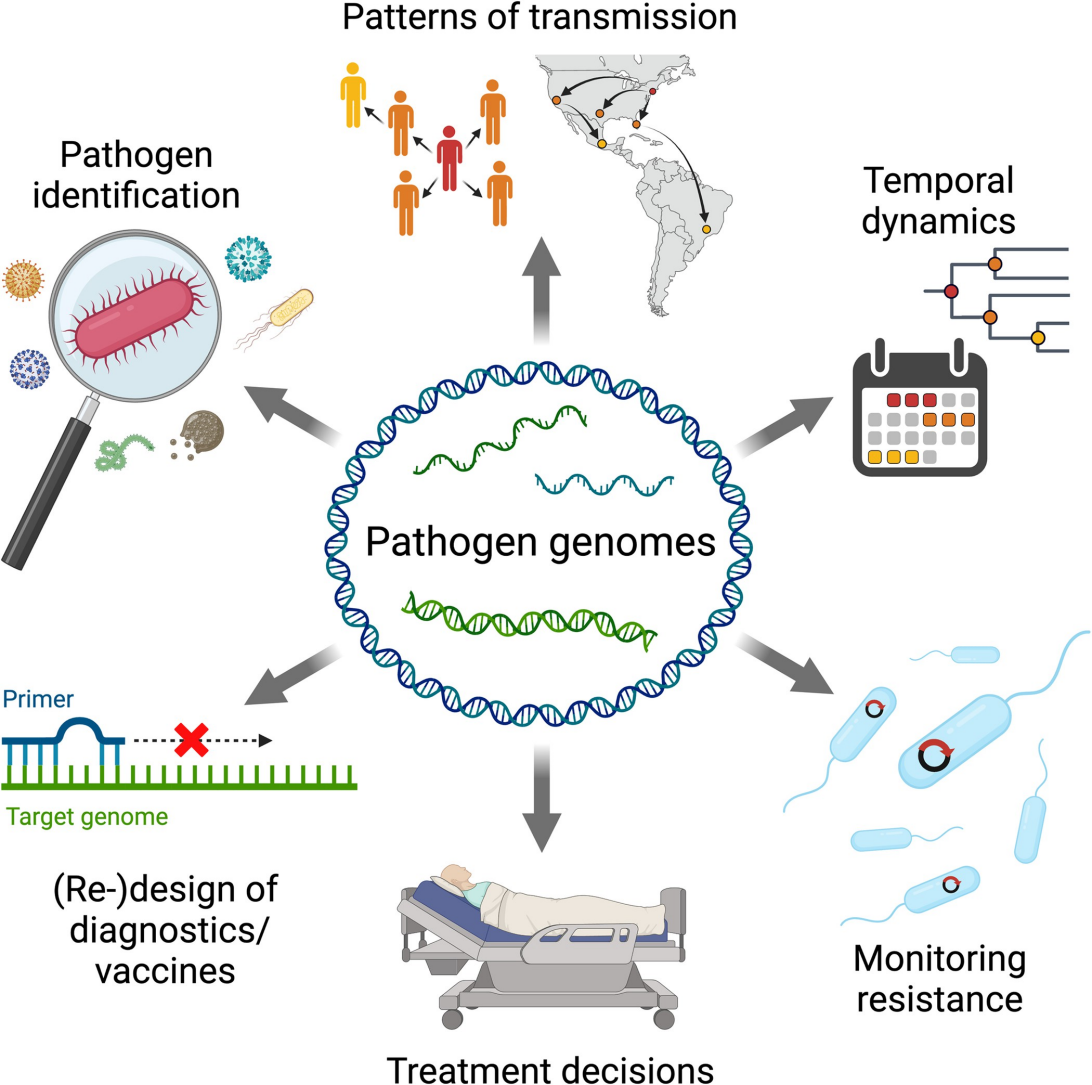
The many uses of pathogen genomes for public health. Created with BioRender.com.

In addition to these important technological advances, pathogen genome sequencing has also risen to prominence due to the many unique ways that pathogen genomes can help us to understand and control the spread of infectious disease. The applications for pathogen genome sequences can generally be assigned to at least one of 3 broad categories, and here, we will discuss several prominent examples from each: (1) the identification and characterization of infectious agents; (2) tracking the movement and evolution of pathogens through space and time; and (3) informing treatments and interventions (Fig 2).

The genome serves as the hereditary material for all forms of life, and as such, each pathogen’s genome encodes a unique set of instructions that can be exploited for unambiguous identification, especially when sequenced in its entirety. In contrast, previous methods for pathogen identification were often based on indirect measures of the genetic code (e.g., phenotypes in culture, complement fixation, restriction fragment length polymorphisms by pulsed-field gel electrophoresis) or small pieces of the genome (e.g., multi-locus sequence typing). These methods are often time intensive, require distinct reagents/approaches for different groups of pathogens, and can sometimes lead to ambiguous or misleading diagnoses. Therefore, full-genome sequencing has emerged as a powerful approach for quickly identifying the causative agent of an infectious disease, and it can be applied in a manner that is largely agnostic to the nature of the pathogen (i.e., metagenomics). For example, in 2013 metagenomic sequencing was used to diagnose a young patient with neuroleptospirosis, thus enabling appropriate intervention with intravenous antibiotics, despite the fact that traditional clinical assays for infectious diseases were all negative [22]. Similarly, in 2014 high-throughput sequencing was used to definitively identify Ebola virus as the cause of a disease outbreak in Guinea [23]. Prior to this, Ebola virus had not been observed outside of a few countries in Central Africa.

Genome sequences can also be used to reconstruct chains of transmission, and therefore, genomic analyses can inform public health initiatives focused on minimizing the spread of infectious disease. Because genomes serve as the hereditary material, any genomic mutations or rearrangements will be inherited from parent to offspring and variants that arise within one infection can be transmitted to a new host. This means that cases from the same outbreak/transmission chain are expected to be caused by genetically identical or very similar pathogens and that genetic divergence between infection-derived genomes will be correlated to epidemiological distance. For example, whole-genome sequences have become instrumental for investigations of bacterial foodborne disease outbreaks through initiatives such as PulseNet [24,25] and GenomeTrakr [26,27]. By providing greater strain resolution than traditional approaches (e.g., pulsed-field gel electrophoresis), whole-genome sequences can more accurately identify cases linked to the same outbreak and pinpoint the initial source of contamination, thus facilitating targeted remediation [28,29]. Similarly, whole-genome sequencing has improved public health interventions for tuberculosis by more accurately identifying recent human-to-human transmission events [30]. Genome sequences have also been used to reconstruct HIV-1 transmission networks to enable targeted public health interventions [31] and have even played an important role in confirming atypical modes of transmission, like the sexual transmission of both Ebola [32] and Zika [33] viruses. In combination with traditional epidemiological investigations, the generation of nearly identical virus genomes from semen samples from the male partners and blood samples from the female partners made sexual transmission the most likely scenario in both cases.

Within the genomes of pathogens, mutations also tend to accumulate at a broadly regular rate through time. This is commonly referred to as a molecular clock, which can be used to estimate dates for important outbreak-related events. Even before high-throughput sequencing enabled routine pathogen sequencing, virus genomes (generated through PCR and Sanger sequencing) were used to help understand the origin of the 2009 swine flu pandemic. Molecular clock analysis demonstrated that the pandemic strain circulated undetected for several months in humans and several years in swine, thus indicating the need for more systematic surveillance for novel influenza viruses [34]. In recent years, genome sequencing and molecular dating analyses have become a routine part of outbreak investigations and have shed light on the emergence of many viruses, including MERS coronavirus [35], Ebola virus [36], HIV-1 [37,38], and Zika virus [39]. Molecular clock analyses have also been used to understand the evolutionary histories and geographical spread of bacterial pathogens, although there can be complications due to high levels of recombination [40] and distinct life-history stages with different rates of evolution (e.g., spore-forming bacteria) [41]. For example, genomes generated using high-throughput sequencing have been used to understand the ancient origins of *Mycobacterium tuberculosis* [42,43], as well as the recent origins of epidemic clones of multidrug-resistant *S*. *aureus* [44].

Pathogen genome sequencing also plays an important role in the contemporary design (and redesign) of diagnostics and vaccines. Many of our current diagnostics are based on the detection of pathogen genomes, and the sensitivity of these diagnostics depends on sequence complementarity between the target pathogen and the assay’s primers/probes, while specificity depends on a lack of complementarity with off-target, near neighbors. For pathogens with high mutation rates, like viruses, it is critical to monitor genome diversity through space [45] and over time [46] to maintain a good match between pathogen and diagnostic. For example, several commercial SARS-CoV-2 diagnostics have lost sensitivity over time (i.e., started generating false negatives) due to evolution of the virus [47,48]. For pathogens with larger genomes and flexible gene content, like bacteria, it is critical to identify genomic targets that are highly conserved and specific to the pathogenic strains of interest [49]. For example, detection of the biothreat agent, *Francisella tularensis*, has been plagued by false positive detection due to a lack of genomic understanding of unculturable, yet related environmental species [50]. Similarly, for a vaccine to be protective, there must be a good match between the antigens included in the vaccine and those expressed by the circulating form of the pathogen. Whole-genome sequencing is a routine part of influenza virus surveillance, used to monitor both the evolution of known strains and the emergence of new reassortants, and each year’s vaccine strain is selected based on these genome sequences [51]. The development of bacterial vaccines can also be aided by genomic sequencing, as regional variation in strains could affect the choice of appropriate antigens. For example, colonization factors in enterotoxigenic *E*. *coli* are diverse, easily detectable by whole-genome sequencing, and are the major components of some ETEC vaccines [52], guided by the regional dominance of specific genotypes.

Genome sequencing can also be used to monitor the ongoing evolution of pathogens for escape from existing therapeutics and to inform the design of new treatments. Antibiotics are our primary tool for fighting bacterial infections, but the pace of antibiotic discovery has slowed considerably and antibiotic-resistant strains of bacteria are emerging at alarming rates. Whole-genome sequencing can be used to accurately predict antimicrobial resistance profiles from sequence data for many bacteria [53], including *M*. *tuberculosis* [54]. As software to perform bacterial genome-wide association studies, powered by machine learning algorithms, become more powerful, genome sequencing will represent an important tool for monitoring resistance at the population level [55] and informing patient-level treatment decisions [56]. Genome sequencing has also become a critical component in the development of one of the most promising alternatives to antibiotics: bacteriophage therapy. High-throughput sequencing is used to screen bacteriophage genomes for deleterious markers (e.g., toxins) and to detect contamination within laboratory stocks [57]. For many years now, genome sequencing has also been a recommended component of the WHO’s strategy for preventing and monitoring drug resistance in HIV [58], and in recent years, there has been a concerted effort to transition to the use of high-throughput sequencing for HIV surveillance because it can detect drug-resistant variants present at low frequency within an infected individual [59].

### Pandemic-driven advances

With the technical foundations and broad utility already established, the public health and research communities were well positioned to rapidly apply pathogen genome sequencing to help understand and respond to the COVID-19 pandemic that began late in 2019. For example, during the very first weeks of the outbreak, unbiased high-throughput sequencing was used to identify and characterize the novel coronavirus that would eventually be named SARS-CoV-2 [60]. These initial genome sequences were publicly released and they allowed for the rapid development of targeted diagnostics and vaccines [61]. They also enabled the design of nucleic acid enrichment strategies specific for SARS-CoV-2 (e.g., tiled amplicon primer sets) [62], which facilitated routine genome sequencing directly from clinical samples.

**Fig 3:**
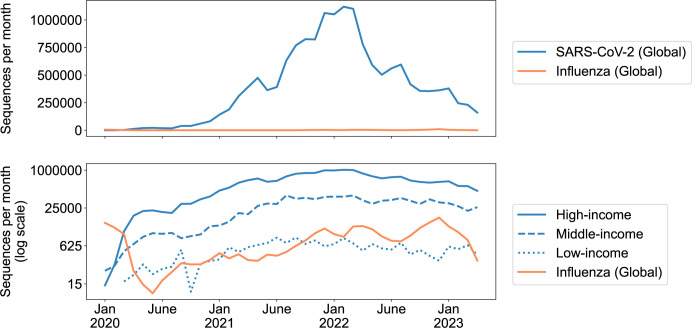
SARS-CoV-2 and influenza virus genomes uploaded each month to GISAID from January 2020 to April 2023. Data last accessed on 2023-05-09. SARS-CoV-2 data obtained from EpiCoV “Global by month” download. Influenza virus data obtained from https://gisaid.org/influenza-subtypes-dashboard/. In the lower panel, SARS-CoV-2 sequences have been divided according to World Bank income categories, with lower-middle and upper-middle combined into a single category (https://datahelpdesk.worldbank.org/knowledgebase/articles/906519-world-bank-country-and-lending-groups). Data underlying this figure can be found in Supporting Information (S1 Data).

Ultimately, pathogen genomic surveillance was implemented at an unprecedented scale in response to the COVID-19 pandemic. In fact, as of May 9, 2023, 15,532,821 SARS-CoV-2 genome sequences had been submitted to the GISAID database (Fig 3). This is several orders of magnitude higher than the number of genomes generated in response to previous outbreaks caused by emerging viruses (e.g., approximately 2,000 Ebola virus sequences from West Africa from 2013 to 2016; less than 1,000 Zika virus sequences from the Americas from 2015 to 2016), and it has even surpassed the total number of available influenza virus genomes (<1 M), for which genomic surveillance programs have existed for more than a decade (Fig 3). The number of contributing sequencing facilities has also been unprecedented. As of May 11, 2023, 222 different countries/territories and >5,700 “submitting labs” have contributed SARS-CoV-2 genomes to GISAID, and many of the sequences of greatest consequence for the public health response have been generated by labs in the Global South [63,64]. Although capacity for high-throughput sequencing was already on the rise prior to the emergence of SARS-CoV-2, the pandemic led to considerable investment in sequencing facilities and genomic surveillance, and SARS-CoV-2 genomes have been used in a variety of ways, including: (1) to understand the origin of the pandemic [65]; (2) to reconstruct transmission chains [66,67]; (3) to monitor the emergence of new variants [63,64,68]; (4) to design and redesign diagnostics and vaccines [69–71]; and (5) to make informed patient treatment decisions (e.g., which monoclonal antibody therapeutics are likely to be effective) [72–74].

In response to the COVID-19 pandemic, there has been a proliferation of software tools aimed at facilitating rapid interpretation and discussion of pathogen genome data. For example, pangolin [75] and nextclade [76] both allow users to quickly assign SARS-CoV-2 genomes to lineages using a dynamic and non-stigmatizing nomenclature, thus providing a consistent and precise vocabulary for the discussion of SARS-CoV-2 genomes [77]. Similarly, web-based “dashboards” have quickly become indispensable tools that have helped to solve 2 important challenges in genomic surveillance: (1) the real-time analysis of genomic data; and (2) the rapid and widespread dissemination of results. For pathogen genomes to be of use during an active outbreak, sequences must be analyzed rapidly and results must be communicated to the wide variety of groups and individuals making public health decisions. Through the use of automated workflows, SARS-CoV-2-focused dashboards like NextStrain’s ncov [78], CoV-Spectrum [79], COG-UK-ME [80], and many others have facilitated continuous, real-time analysis of virus genomes throughout the pandemic. They have also helped democratize access to genomic epidemiology. Not only did these dashboards facilitate the real time sharing of results, but also because of the interactive nature of many of these websites, they enable users to parse the available data in customized ways, even if they do not have expertise in genomics, and with a minimal investment of time. In many cases, the code base underlying these dashboards is also open source and contributions from the community are welcome. This approach not only enhances transparency, but also facilitates the adaptation of these resources to other pathogens, outbreaks, and applications.

The unprecedented magnitude of the COVID-19 pandemic (and the associated sequencing response) has also driven the development of novel tools and methods focused explicitly on the analysis and visualization of very large datasets (i.e., containing millions of sequences). While extremely powerful, the tools that existed at the start of the pandemic (e.g., NextStrain, BEAST) were designed to process datasets with, at most, a few thousand genome sequences. For an intensely sequenced pathogen like SARS-CoV-2, this means that datasets need to be substantially downsampled prior to analysis. And while there are tools that facilitate downsampling in ways that aim to minimize bias [11,81], downsampling is not appropriate for all applications and its impact is usually not rigorously evaluated [82]. One example of a novel tool that has facilitated comprehensive phylogenetic analyses for SARS-CoV-2 is UShER [83]. Rather than following the traditional approach for building phylogenies, which starts from scratch each time new data is acquired, UShER adds new sequences to existing trees, and it does so quickly and with high accuracy. Not only is this approach well suited to active outbreaks, where new sequences are being generated regularly, but it also scales efficiently and therefore can add new data to trees containing millions of sequences within an actionable timeframe [84]. Another important tool for enabling comprehensive phylogenetics of SARS-CoV-2 is Taxonium [85], which is optimized for visualizing and exploring trees that contain millions of sequences. And although SARS-CoV-2 was the impetus for the development of these tools, they are not SARS-CoV-2 specific. Both have already been applied to other high-priority pathogens, and tools like these are likely to become more widely needed as the level of pathogen sequencing continues to increase.

Another major challenge during the response to global health emergencies is facilitating communication and data sharing between the many relevant groups generating and using pathogen genome sequences (e.g., public health labs, academic research groups, biotechnology companies, governments, and media). At the national level, initiatives like the CDC’s SPHERES (SARS-CoV-2 Sequencing for Public Health Emergency Response, Epidemiology and Surveillance) represent a major advance over previous outbreak responses. SPHERES has utilized modern software tools (e.g., Zoom and Slack) to facilitate regular and active discussions between diverse stakeholders from across the United States [86]. Within the United Kingdom, the COVID-19 Genomics UK (COG-UK) Consortium went even further by not only facilitating discussion, but also actually creating a centralized system for rapidly collecting, processing, and sharing SARS-CoV-2 genome sequences, along with associated sample metadata [87]. This system, powered by the CLIMB-COVID compute infrastructure [88], was able to leverage a distributed network of clinics and sequencing facilities to provide a unified view of the pandemic at the national level [89].

Finally, the COVID-19 pandemic has renewed interest in the use of wastewater sampling for pathogen surveillance, which, when combined with genome sequencing, provides a passive yet powerful approach for tracking the emergence of new viruses and variants. Pathogen surveillance in wastewater dates back to the 1940s, where poliovirus was detected from sewage in New Haven, Connecticut and New York City [90]. Wastewater sampling for SARS-CoV-2 surveillance gained attention due to its comprehensive and unbiased detection capability [91] and recent work has broadened into the detection of influenza virus [92], monkeypox virus [93], and antimicrobial resistance genes [94]. Wastewater surveillance has also recently been used again to track poliovirus, this time identifying circulation in several non-endemic regions, with the resulting sequences implicating strains from the replication-competent oral poliovirus vaccine [95–97]. One of the challenges for high-resolution surveillance, where the detection of specific mutations is required for genomic epidemiology, is the presence of mixed genotypes. However, recent work suggests that the deconvolution of related viruses is possible due to informatics advances made during the SARS-CoV-2 pandemic [98]. Wastewater is also an attractive sampling matrix for the early identification of emerging pathogens as it is independent of voluntary testing campaigns and can be used as a community forecasting tool [99]. The challenge of wastewater surveillance for new pathogens is that deep metagenomic sequencing is required for novel discovery efforts. As sequencing becomes cheaper or new enrichment approaches become feasible, routine metagenomic surveillance of wastewater samples may be possible to monitor for the emergence of novel viral, bacterial, and fungal pathogens.

### The future of data sharing

Pathogen genome sequences have quickly become an indispensable part of how we prepare for and respond to infectious disease outbreaks, but the benefit of these sequences for public health is highly dependent on timely and equitable sharing of data [100]. Prior to the COVID-19 pandemic, most pathogen genomes were shared through a member of the International Nucleotide Sequence Database Collaboration (INSDC), which is a collection of repositories (DDBJ, ENA, and NCBI) that share a common policy of free and unrestricted data use [101]. In many respects, this represents an ideal system for sharing outbreak-related data because it ensures that the available sequences can be used as broadly as possible, both for research and commercial applications (e.g., the development of diagnostics and vaccines). Unrestricted data sharing through INSDC repositories has also enabled the development of many important data analysis resources for pathogens (e.g., the NIAID’s Bioinformatics Resource Centers, including the Los Alamos HIV sequence database and the Bacterial and Viral Bioinformatics Resource Center, which recently integrated PATRIC, IRD, and ViPR [102]). These resources have facilitated discoveries related to pathogen genomes through expert curation and annotation of raw sequences submitted to INSDC repositories.

However, the INSDC’s approach only works in the context of public health if data producers are comfortable uploading their sequences in real time, which generally means prior to any in depth analysis or publication. Unfortunately, the INSDC’s data use policy is not able to provide any protections for data producers with regard to attribution and/or requirements for collaboration. Therefore, many data producers are hesitant to upload data immediately to the INSDC, fearing that they may get scooped by others using their own data. GISAID was introduced as an alternative to the INSDC model, one that explicitly protects the interests of data producers by requiring that users adhere to a database access agreement [103]. GISAID is run through an independent, nonprofit that was initially established to facilitate the sharing of influenza genomes [61], but, with the onset of the COVID-19 pandemic, GISAID expanded its scope to include SARS-CoV-2 (EpiCoV) (Fig 1).

As a result of these protections, as well as a streamlined submission system, GISAID was widely embraced by the international community during the COVID-19 pandemic, and is particularly popular with data producers in LMICs who may not have the resources to analyze and publish their data as quickly as groups in high-income countries [104]. In many respects, GISAID also appears well primed to further expand in the future. However, several recent controversies have imperiled the trust that GISAID has worked so hard to establish [105–107], and it is clear that substantial changes are needed with regard to the transparency of GISAID governance. GISAID has also failed to deliver on an initial promise to serve only as a temporary repository, with data eventually transferred to the INSDC [103]. In fact, there is currently no direct mechanism for transferring sequences from GISAID to the INSDC. As a result, many viral genome sequences have effectively become siloed in a database that prevents data sharing with unregistered users, and therefore, these sequences cannot be integrated into existing bioinformatics resources that openly share curated sequence datasets (see above).

As we look to the future, our needs with regard to data sharing are pretty clear, though it is less clear exactly how these needs will be met. First, we need to do everything we can to encourage rapid data sharing, and this will have to include protections for the interests of the data providers. Second, the guidelines for data access must be transparent and fairly enforced, and there must be an official process for appealing decisions that result in the loss of access. Third, there must be a streamlined process for transitioning data from a restricted repository to one that allows unrestricted data use. The need to protect the interests of data providers is real, but it is not indefinite. Once the providers have published on their data, it should become freely available for additional use. All of these needs could feasibly be met through cooperation between GISAID, the INSDC, and the broader community of stakeholders (i.e., funding agencies, data providers, and data users). However, if such cooperation does not materialize, then we may need new solutions that can meet all of the requirements needed for seamless and equitable incorporation of pathogen genome sequencing into our global public health response to both epidemic and endemic pathogens [100].

### The future of genomic surveillance

The SARS-CoV-2 pandemic led to the development of exciting new techniques, data sharing platforms, and analytical tools, but it also highlighted important issues, gaps, and inequities that, if addressed correctly, could improve future genomic surveillance efforts and better prepare us for the next public health emergency. For example, massive emergency investments facilitated the development of sequencing infrastructure that has allowed for the mass production, submission, and analysis of pathogen genomes, but as this investment in SARS-CoV-2 sequencing wanes (Fig 3), we are now faced with the challenge of maintaining this infrastructure in the absence of a public health emergency. Fortunately, most of this infrastructure is flexible enough to be applied to many different pathogens of concern, and many infectious diseases have been neglected over the past several years as the world’s attention has been drawn to SARS-CoV-2. Therefore, the key to maintaining our recent advances likely lies in a pivot away from a sole focus on SARS-CoV-2 and towards a more inclusive scope [108]. For example, during the SARS-CoV-2 pandemic, antimicrobial resistant (AMR) bacteria lost focus, but continue to pose a substantial public health threat [109]. Genomic surveillance for many endemic viruses is currently well below optimal levels [110], and our capacity to efficiently diagnose fungal infections and predict antifungal resistance is severely limited [111]. By pivoting to a more inclusive approach to genomic surveillance, including viral, bacterial, and fungal targets, and potentially utilizing multiplex detection and sequencing strategies (Box 1), we can broadly improve public health and maintain existing capacity. If infrastructure is not supported and data sharing pipelines are not maintained, a complete rebuild will be needed for the next pandemic, which will drastically increase response time.

**Box 1. Priority areas for future investment**

1. Open source software development and maintenance.

- To realize the full potential of pathogen genomes for improving public health, we need software that is accurate, easy to use, freely available, and able to quickly deliver actionable results for ever-expanding datasets.
- Despite many recent advances in this area, a lack of appropriate software remains a barrier for broader implementation of pathogen genome sequencing in public health responses, especially for applications outside of the tracking of emerging viruses [112].

Specific needs: New tools to fill gaps and streamline workflows with a priority on interoperability; continued maintenance of existing, high-impact tools (otherwise they will quickly lose their value).

2. Multiplex detection and sequencing strategies.

- To broaden the utility of genome sequencing for public health, it will be important to invest in approaches that are capable of detecting and characterizing multiple pathogens simultaneously.
- If we continue to focus on “singleplex” strategies, our effort will remain heavily biased toward only the highest priority pathogens.

Specific needs: Broader implementation of diagnostic assays (e.g., CRISPR-based nucleic acid detection strategies [113]) and sequencing strategies (e.g., probe-based hybrid capture [114,115]) that can simultaneously detect/characterize multiple pathogens with a single set of reagents.

3. Cost-effective enrichment of large/diverse targets.

- Targeted nucleic acid enrichment strategies are critical for facilitating pathogen genome sequencing directly from clinical samples.
- Options are limited, and often not cost-effective, when assays need to target a large amount of sequence diversity in a single assay, e.g., for multiplex enrichment protocols (see above) or whole-genome sequencing of pathogens with large genomes, like bacteria, which is becoming increasingly important with the adoption of culture independent diagnostic tests [29].

Specific needs: Strategies that can enrich a large variety of nucleic acid targets with a single set of reagents, while remaining affordable enough for routine implementation.

4. Understanding the optimal level of sequencing.

- As we look to the future, it will be important to transition from a perspective of “the more the better,” to one that carefully considers the required level of genome sequencing [116] and optimal sampling strategies [82] for addressing the most critical needs for our public health response.

Specific needs: Quantitative frameworks for evaluating the impact of different approaches and levels of investment in sequencing (e.g., [116,117]); clearly defined objectives for the role of pathogen genomics in preparing for and responding to public health threats.

5. Implementation of passive, long-term surveillance programs.

- Passive sampling methods, such as wastewater surveillance, will be critical to monitor for the presence of novel pathogens or variants.
- We should broaden existing programs (e.g., implement wastewater sampling for arriving aircraft and cruise ships [118], utilize Biowatch program infrastructure for airborne pathogen detection [119]) and ensure that these programs can be continuously operated, over long periods of time.

Specific needs: Standardized sampling and analysis protocols for the detection of specific pathogens; buy-in from funding agencies as well as close collaboration between federal monitors and local laboratory response networks.

Additionally, despite substantial increases in global sequencing and analysis capacity over the last several years, important disparities remain that undermine outbreak preparedness at both local and international scales [108,120,121]. During the pandemic, most of the genomic data was generated in high-income countries (Fig 3), but many variants of concern emerged from LMICs [120,122]. Furthermore, new pathogens can emerge from anywhere and quickly spread around the globe. Therefore, our future genomic surveillance strategy must involve expanding capacity in LMICs. This will likely require increases in local investments for public health initiatives [108,121,123], as well as continued support through international public–private partnerships, such as the Africa Pathogen Genomics Initiative. Fortunately, there are many existing regional centers of excellence and support networks that can help, not only to establish new sequencing centers, but also to provide the ongoing support needed to sustain and grow these programs [123–125].

Looking forward, it will also be important to carefully consider the limitations of genome sequencing, which will help us focus our efforts in ways that will optimize the return on investment for public health. Despite unprecedented sequencing efforts during the pandemic, we still sequenced a small fraction of the total number of SARS-CoV-2 infections and the turnaround time between sample collection and genome submission was often >3 weeks [120]. Therefore, in practice, genome sequences were not informative for many of the most time-sensitive public health decisions, like the implementation of border closures; by the time a new variant of concern was identified, it was likely already geographically widespread. Technological advances are likely to decrease sequencing turnaround times in the future [126], but we would still need to be sequencing a very large number of samples each day to detect a new variant with high probability prior to substantial community spread [116]. It is also challenging to infer the functional consequences of mutations from genome sequences in isolation. Rather, it is the change in prevalence over time that is most powerful for the identification of variants of concern [63,127]. Therefore, pathogen sequencing is most likely to be beneficial for addressing questions that will remain relevant over longer timescales (e.g., forecasting future surges in cases, redesigning diagnostics and vaccines, selecting the most appropriate treatment regimens).

Although we have benefitted in many ways from genome sequencing during the SARS-CoV-2 pandemic, it is also true that, in some respects, there were diminishing returns on investment as more and more cases were sequenced, especially from similar locations and points in time. At one extreme, we were able to realize several benefits with just a single genome sequence, including the initial identification of the causal agent of COVID-19 and the information needed to initiate the design of vaccines and diagnostics. At the other end of the spectrum, is the use of pathogen genomes to identify and track the spread of new variants. In this case, more genomes means earlier detection of new variants and more accurate estimation of variant frequencies [116]. There are also instances in which comparable information could likely have been obtained from sub-genomic analyses. For example, most of the mutations that have been shown to impact SARS-CoV-2 infectivity and immune evasion are located in the Spike glycoprotein gene. This protein is also the only antigen contained in most vaccines currently in use against SARS-CoV-2. By focusing our sequencing efforts on high-priority genomic regions, like the SARS-CoV-2 Spike, we may be able to decrease costs per target pathogen while maintaining most (though not all) of the utility of the generated sequences [128]. Therefore, as we prepare for future outbreaks, we need to carefully consider the optimal sequencing effort that will ensure a balance between the associated costs and the resulting benefits (Box 1). This will not only require the establishment of quantitative frameworks for evaluating the impact of different investments in sequencing (e.g., [116,117]), but also a clearly defined set of objectives for the role of pathogen genomics in preparing for and responding to public health threats.

Given the massive growth in the size of the sequencing community and the need for rapid turnaround of data, we also face important challenges regarding workflow standardization, quality assurance, and the dissemination of results. Standardization will always be a challenge when 100s to 1,000s of groups are simultaneously contributing to a field of study. However, standardization tends to arise organically whenever high-quality resources are provided that are free of charge, easy to use, and do not require any loss of data ownership. Great examples during the SARS-CoV-2 pandemic include the ARTIC Network primers for genome amplification [62], the Pangolin software for lineage naming [75], and the NextStrain platform for phylogenetic analysis [11]. It is important to continue to invest in efforts like these, as they must be actively maintained to remain relevant, and should be expanded to cover other high-priority pathogens (Box 1). For example, to keep pace with virus evolution, multiple versions of the ARTIC amplicon panel had to be developed over the course of the pandemic to address the dropout of genomic regions due to primer mismatch [129]. We also need new software pipelines tailored specifically for analysis of pathogens with larger, more complex genomes, like bacteria, fungi, and even some dsDNA viruses (Box 1) [112,130]. And finally, we must invest in robust, automated protocols that can facilitate sequence curation in a sustainable way to ensure data quality and therefore also the quality of downstream interpretations.

Over the last couple decades, technological advances have enabled the routine sequencing of pathogen genomes. Combined with a growing and highly engaged community of scientists, this has revolutionized the way we study and respond to outbreaks of infectious disease, and as we transition out of a period dominated by the emergency response to the COVID-19 pandemic, we are well placed to broadly apply the benefits of routine genome sequencing to the full diversity of human pathogens.

## Supporting information

S1 Data Data underlying the graphs in Fig 3. Monthly SARS-CoV-2 genomes uploaded to GISAID [“SARS-CoV-2 Seqs (Global)”] were obtained from the EpiCoV “Global by month” download. Monthly influenza virus genomes uploaded to GISAID [“Influenza Seqs (Global)”] were obtained from https://gisaid.org/influenza-subtypes-dashboard/. In the lower panel of Fig 3, SARS-CoV-2 sequences have been divided according to World Bank income categories, with lower-middle and upper-middle combined into a single category [“SARS-CoV-2 Seqs (Middle-income)”].
(TSV)

## Abbreviations

AMR: antimicrobial resistant
COVID-19: Coronavirus Disease 2019
HIV: human immunodeficiency virus
INSDC: International Nucleotide Sequence Database Collaboration
LMIC: low-and-middle-income country
SARS-CoV-2: Severe Acute Respiratory Syndrome Coronavirus 2
